# Binocular visual function of Japanese eyes with nondiffractive extended depth-of-focus intraocular lenses made of high water-content hydrophobic acrylic material

**DOI:** 10.1007/s10384-025-01300-5

**Published:** 2025-11-04

**Authors:** Hisaharu Suzuki, Yuka Ota, Seiichiro Hata, Keiichiro Minami, Hiroko Bissen-Miyajima

**Affiliations:** 1 Zengyo Suzuki Eye Clinic, Fujisawa, Japan; 2 Department of Ophthalmology, Tokyo Dental College Suidobashi Hospital, Kandamisaki-cho 2-9-18, Chiyoda-ku, Tokyo 101-0061 Japan; 3 Yokohama Sky Eye Clinic, Yokohama, Japan

**Keywords:** Binocular visual function, Japanese eye, Nondiffractive extended depth-of-focus IOL

## Abstract

**Purpose:**

To prospectively evaluate bilateral visual function in Japanese patients implanted with nondiffractive extended depth-of-focus (EDoF) intraocular lenses (IOLs).

**Study design:**

Multisite prospective observational study.

**Patients and methods:**

This study included 48 eyes of 24 Japanese patients with cataracts (mean age: 68.7 ± 9.2 years) who underwent bilateral implantation of nondiffractive EDoF IOLs CNAET0 (Clareon Vivity, Alcon), made of high-water-content hydrophobic acrylic material. Three months postoperatively, binocular uncorrected and distance-corrected visual acuities (BUCVA and BDCVA) were assessed at far, 66 cm, and 40 cm. Binocular photopic contrast sensitivity (CSV-1000), binocular defocus curves, spectacle independence, and photic phenomena (glare, halo, starburst, and waxy vision) were also assessed.

**Results:**

The mean logMAR BUCVA/BDCVA was –0.11/–0.15 at far, 0.05/0.08 at 66 cm, and 0.18/0.22 at 40 cm. Binocular contrast sensitivity was within the normal range for individuals aged 60–69 years across all spatial frequencies. The mean defocus curve demonstrated 0.1 logMAR or better between −2.0 and +1.0 D addition, with better performance of 0.0 logMAR or better between −1.5 and +0.5 D addition. All patients were spectacle-independent for distance and intermediate vision, whereas nine of the 24 patients (37.5%) required spectacles for near vision. None of the patients reported severe photic phenomena; 17 patients (70.8%) did not experience glare or starburst, and 20 patients (83.3%) did not report halo or waxy vision.

**Conclusion:**

Bilateral implantation of nondiffractive EDoF IOLs provided good binocular functional vision from far to near, although some patients may require spectacles for near vision. The photic phenomenon was minimal.

## Introduction

Among presbyopia-correcting intraocular lenses (IOLs), extended depth-of-focus (EDoF) IOLs are designed to minimize spectacle dependence for distance and intermediate vision, whereas trifocal IOLs provide coverage across all distances [1]. There are 2 types of EDoF IOLs: diffractive and nondiffractive. The Symfony (Johnson & Johnson Surgical Vision) is a diffractive EDoF utilizing echelette gratings on the anterior surface to add +1.75 D for an extended range of vision from 0.7 m to far, as well as compensating for chromatic aberration in distance vision [2]. Although good visual outcomes are obtained at far and intermediate distances, some patients experience photic phenomena, such as starbursts and glare [3], because of optical disturbances from the added power [4]. The AcrySof Vivity (Alcon) is a nondiffractive EDoF IOL that modifies the wavefront across its 2.2-mm central optic through a slightly elevated plateau on the anterior surface and altered posterior curvature [4]. This design provides good quality of vision from far to intermediate distances with fewer photic disturbances than those of diffractive EDoF IOLs [3, 5]. In Japan, this nondiffractive EDoF was introduced as Clareon Vivity in 2023. Clareon is made of high water-content hydrophobic acrylic material as compared with AcrySof, and its superiority has been experimentally validated [6]. The clinical outcomes of Clareon monofocal and Clareon trifocal IOLs were evaluated, showing results comparable to those of AcrySof [7, 8]. Although similar results were expected with the Clareon Vivity, its clinical outcomes have not yet been reported. Therefore, this prospective multicenter study aimed to evaluate binocular visual function after implantation of Clareon Vivity IOLs in Japanese patients.

## Patients and methods

### Participants

This investigator-initiated prospective study was approved by the local institutional review board (MINS IRB, Tokyo, Japan) and was conducted in accordance with the tenets of the Declaration of Helsinki. Written informed consent was obtained from all the patients before enrollment. Patients who underwent bilateral cataract surgery with implantation of nondiffractive EDoF IOLs were recruited from 3 sites: Zengyo Suzuki Eye Clinic (Fujisawa, Japan), Yokohama Sky Eye Clinic (Yokohama, Japan), and Tokyo Dental College Suidobashi Hospital (Tokyo, Japan). The inclusion criteria were corneal astigmatism of 0.75 D or less, a target refraction of emmetropia, and potential postoperative corrected visual acuity of 20/30 or better. Patients with other ocular diseases influencing visual function, irregular corneal astigmatism, a history of intraocular or corneal surgery, or other systemic or ophthalmic diseases that were unsuitable for this study were excluded.

### Sample size determination

The sample size was determined to evaluate binocular distance-corrected visual acuity. In a previous study using AcrySof Vivity IOLs [5], the mean binocular-corrected distance visual acuity was –0.028 (standard deviation [SD]: 0.084) in logarithm of the minimum angle of resolution (logMAR) units. The minimum step in the Landolt ring charts was 0.05 logMAR. To detect a difference of 0.07 logMAR, a sample size of 18 patients was required, with a significance level of .05 and a detection power of 90% (R version 3.6.1, package ‘pwr’ version 1.3). Considering the difficulty of participant recruitment without toric models and a dropout rate of 15%, the target sample size was determined to be 30 patients.

### Intraocular lenses and surgery

CNAET0 (Alcon) is a single-piece, hydrophobic acrylic, nondiffractive EDoF IOL, identical to DFT015 (Alcon) except for its material composition. The modified material incorporates hydroxyethyl methacrylate instead of phenylethyl methacrylate, with a water content of 1.5% at 35º C and glass transition temperatures of 9.1 °C. The refractive index was 1.55 at 35 °C. The IOL optics were aspheric, with a diameter of 6 mm and a sharp edge on the posterior surface, and the wavefront was modified in the central 2.2-mm area. The power of all the IOLs was determined for emmetropia by use of biometry, and a power calculation formula was routinely used at each site.

The cataract surgeries were performed by a single, experienced surgeon at each site. Cataracts were removed by use of phacoemulsification and aspiration techniques through 2.2- to 2.4-mm-wide temporal corneal incisions, and the IOLs were inserted into the capsular bag by use of specific injectors.

### Postoperative examinations

Binocular visual acuity, binocular contrast sensitivity, and defocus curves were examined 3 months postoperatively.

The binocular uncorrected and distance-corrected visual acuities (BUCVA and BDCVA, respectively) at distances of 5 m, 66, and 40 cm were measured by use of Landolt ring charts under photopic illumination (85–110 cd/m^2^). The manifest refraction spherical equivalent (MRSE) was also measured during monocular distance-corrected visual acuity (DCVA) at 5 m and used for the BDCVA examination. Visual acuity was converted to logMAR for subsequent analyses. Binocular contrast sensitivity was measured by use of a CSV-1000 (Vector Vision) under distance corrected with the measured MRSE and photopic illumination (85 cd/m^2^). Logarithmic contrast sensitivities were obtained at spatial frequencies of 3, 6, 12, and 18 cycles per degree (cpd). Binocular defocus curves between –3.0 and +1.5 D were measured in increments of 0.5 D.

The participants were asked about their use of spectacles and symptoms of photic phenomena, such as glare, halos, starbursts, and waxy vision. The severity of each symptom was graded on a 4-point scale: absent, mild, moderate, and severe.

### Statistical analysis

MRSE is defined as the best correction at infinity. Because MRSE measured at 5 m typically shows a –0.2 D shift, this value was adjusted to obtain the MRSE at infinity [9]. For example, MRSE of –0.5 D at 5 m corresponds to –0.7 D at infinity. The means and SDs of the clinical outcomes were obtained. The means of BUCVA and BDCVA at each distance were compared by use of the Wilcoxon signed rank test. The distributions of MRSE, along with the cumulative percentages of eyes achieving BUCVA and BDCVA at each distance, were reported in a standard manner. Additionally, for BDCVA at 5 m, the 95% CI was calculated for comparison with the AcrySof Vivity IOLs [5].

## Results

Twenty-six participants were enrolled in the study, and 2 participants dropped out—one because of surgery cancellation and the other because of a change in the IOL model. Thus, 48 eyes of 24 participants (8 men and 16 women) were evaluated and deemed eligible for analysis. The demographic data are listed in Table 1. During the postoperative period, no degradation of IOL clarity due to glistenings was observed in any of the eyes during slit-lamp microscopy examinations.

**Table 1:** Participants’ demographic data

	Mean (SD)	Range
Age, y	69.7 (9.2)	54–85
Height, cm	159.0 (7.9)	145–177
Axial length, mm	24.5 (1.3)	22.3–27.3
Mean keratometric power, D	43.81 (1.00)	42.13–45.88
Corneal astigmatism, D	0.45 (0.19)	0.00–0.75
Power of implanted IOLs	18.1 (3.5)	10.5–23.5

**Fig. 1:**
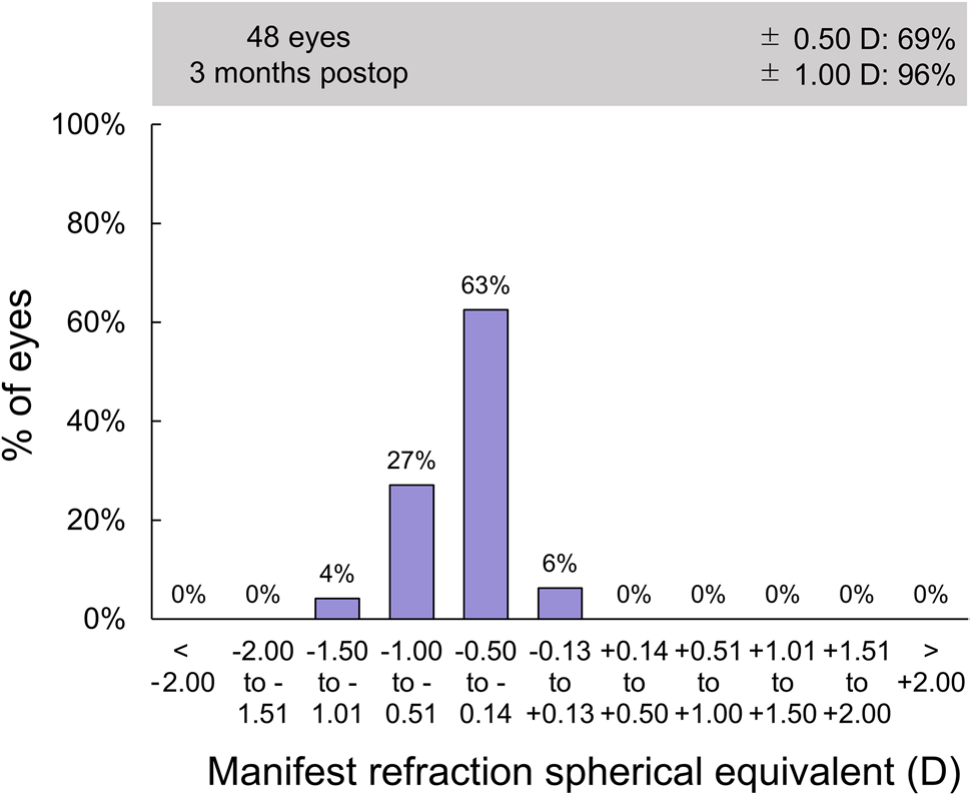
Distribution of manifest refraction spherical equivalent (MRSE) of eyes with nondiffractive extended depth-of-focus intraocular lenses

The mean monocular DCVA was –0.13 (SD: 0.08) logMAR, and all eyes obtained –0.00 logMAR or better. The mean MRSE at infinity was –0.39 (SD: 0.26), which was shifted to myopic in the range of –1.20 to 0.00 D. Figure 1 shows the distribution; 69% and 96% of eyes were within ±0.5 D and ±1.0 D, respectively.

**Fig. 2:**
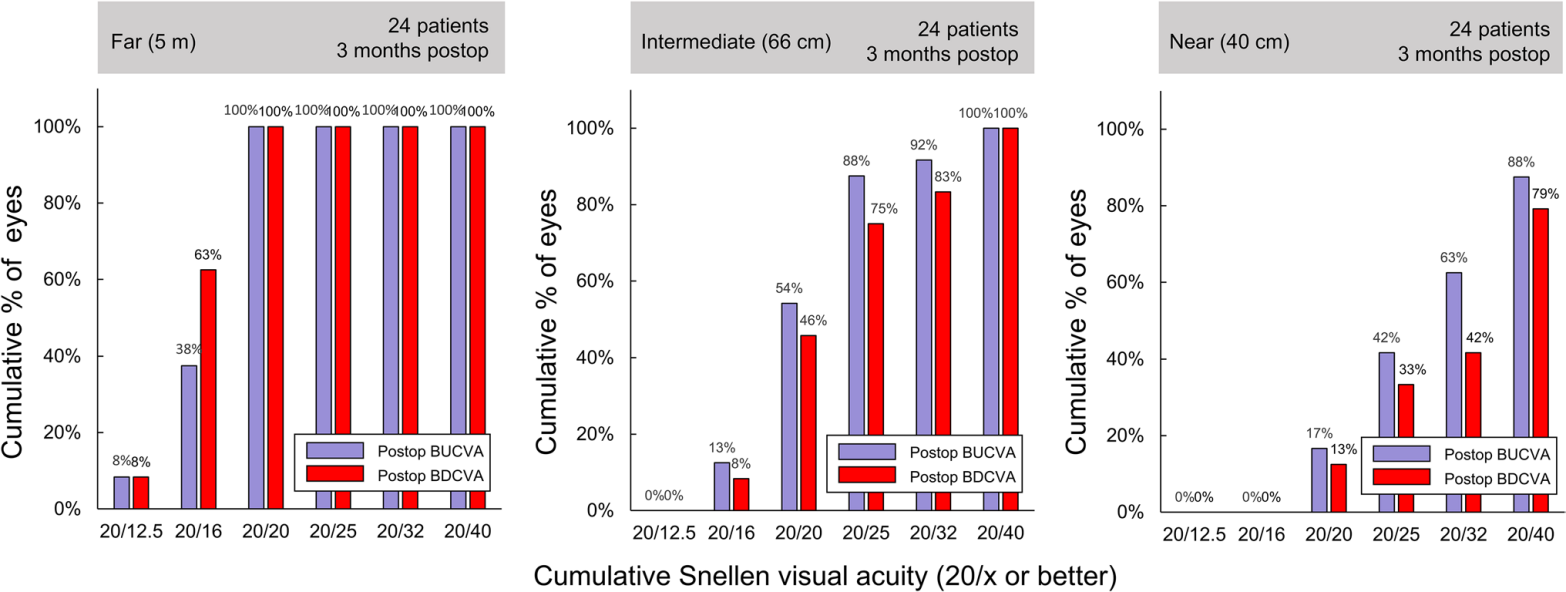
Cumulative percentage of patients achieving binocular uncorrected and distance-corrected visual acuities (BUCVA and BDCVA, respectively) at distances of 40 cm (near, right), 66 cm (intermediate, center), and 5 m (far, left) with nondiffractive extended depth-of-focus intraocular lenses

Table 2 lists the postoperative BUCVA and BDCVA at each distance. The mean BDCVA at 5 m was significantly better than the BUCVA (*P* = .011, Wilcoxon signed rank test). In contrast, at 40 cm, the mean BUCVA exceeded the mean BDCVA (*P* = .0075), that could be attributed to myopic MRSE. The 95% CI of BDCVA at 5 m was between –0.18 and –0.12 logMAR, which was not inferior to the results obtained with the AcrySof Vivity [5]. Figure 2 shows the cumulative percentages of patients achieving BDCVAs and BUCVAs at distances of 5 m (far), 66 cm (intermediate), and 40 cm (near). A postoperative BDCVA of 20/20 or better was obtained in all the participants at far distance. At intermediate and near distances, BUCVA was better than BDCVA owing to myopic shift in the MRSE (Fig. 1).

**Table 2:** Postoperative binocular uncorrected (BUCVA) and distance-corrected (BDCVA) visual acuities at distances of 5 m, 66 cm, and 40 cm

	Mean (SD)	Range
BUCVA, logMAR		
at 5 m	− 0.11 (0.08)	− 0.30 to 0.00
at 66 cm	0.05 (0.10)	− 0.11 to 0.30
at 40 cm	0.18 (0.14)	0.00–0.52
BDCVA, logMAR		
at 5 m	− 0.15 (0.07)	− 0.30 to 0.08
at 66 cm	0.08 (0.12)	− 0.11 to 0.30
at 40 cm	0.22 (0.16)	0.00–0.52

*logMAR* logarithm of the minimum angle of resolution

**Fig. 3:**
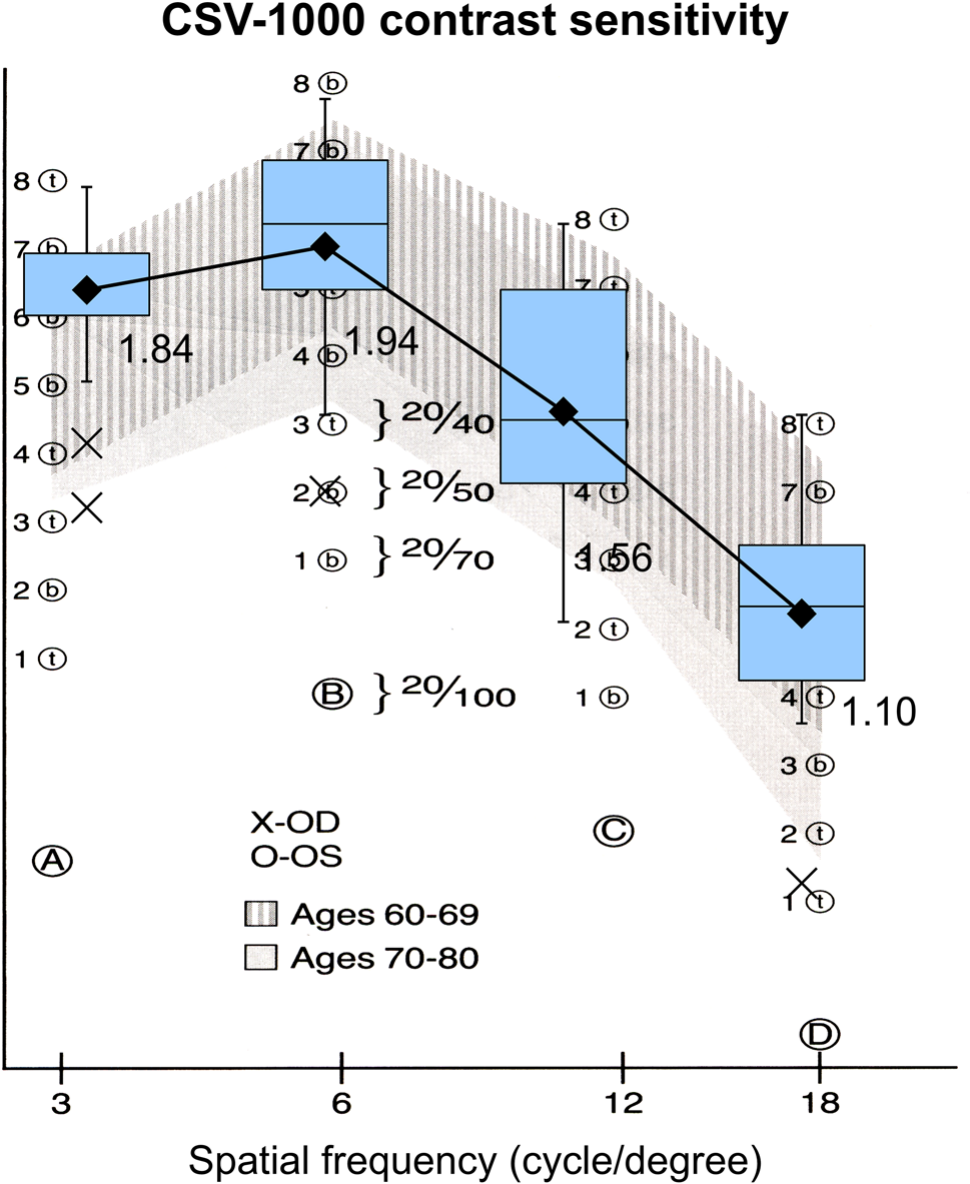
Binocular contrast sensitivity of patients with nondiffractive extended depth-of-focus intraocular lenses

**Fig. 4:**
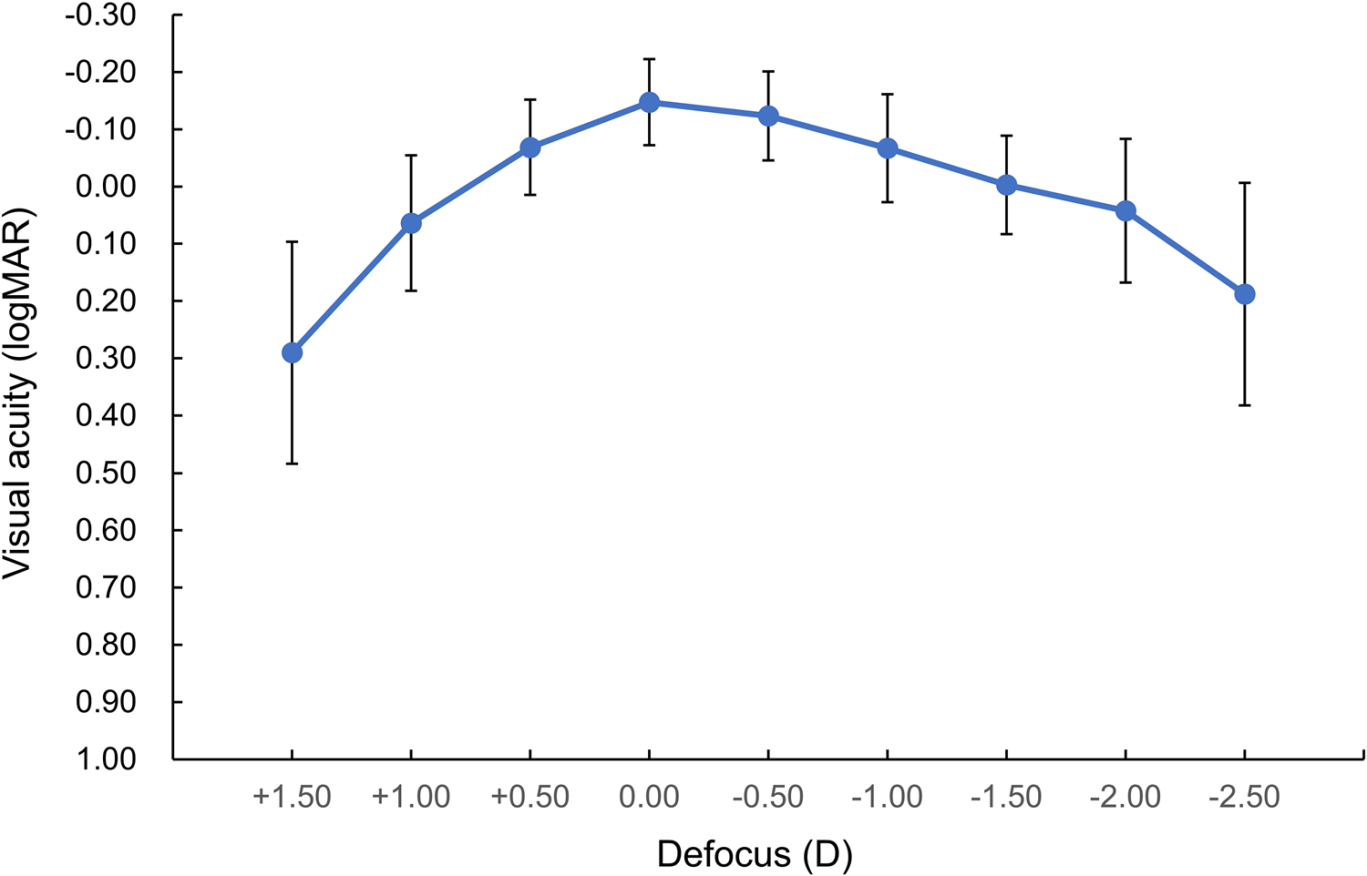
Binocular defocus curve of patients with nondiffractive extended depth-of-focus intraocular lenses

The mean binocular photopic contrast sensitivities were within the normal range for all spatial frequencies (Fig. 3). Figure 4 shows the binocular defocus curve, demonstrating that BUCVA of 20/20 or better could be obtained between –1.50 and +0.5 D.

All the patients were spectacle-independent for distance and intermediate vision; however, nine of the 24 patients (37.5%) required spectacles for near vision when needed, such as for reading fine print. Table 3 lists the number of participants reporting subjective symptoms and their corresponding severity levels. None of the patients reported experiencing severe photic phenomena. Seventeen patients (70.8%) did not experience glare or starbursts, and 20 patients (83.3%) did not report halos or waxy vision.

**Table 3:** Numbers of participants reporting subjective symptoms and their severity

Symptom	Absent	Mild	Moderate	Severe
Glare	17 (70.8%)	5 (20.8%)	2 (8.3%)	0 (0.0%)
Halos	20 (83.3%)	3 (12.5%)	1 (4.2%)	0 (0.0%)
Starburst	17 (70.8%)	5 (20.8%)	2 (8.3%)	0 (0.0%)
Hazy or blurry vision	20 (83.3%)	3 (12.5%)	1 (4.2%)	0 (0.0%)

## Discussion

This study demonstrates that the bilateral implantation of a nondiffractive EDoF IOL made of Clareon material in Japanese patients provides good binocular visual function for far and intermediate distances, without compromising contrast sensitivity or causing severe photic phenomena. Table 4 compares the BDCVA at far, intermediate, and near distances with results previously obtained from patients in the European Union, the United States, and India following the implantation of AcrySof Vivity IOLs [3, 10–12]. Despite differences in IOL materials and patient demographics, our findings were comparable to those of these previous reports. Similarly, previous studies found no significant differences in visual outcomes between Clareon and AcrySof materials for monofocal [7, 13] and trifocal [8, 14] IOLs, supporting the equivalence of Clareon Vivity IOLs with earlier AcrySof models. Photic phenomena following the implantation of EDoF IOLs are expected to be lower than those of diffractive multifocal IOLs. According to a report on trifocal IOLs using the same questionnaire for photic phenomena and severity scale as in this study, 14% to 32% of the patients reported no glare or halos [8]. In contrast, over 70% of the patients with this lens reported no such photopsia, indicating a markedly lower incidence of these visual disturbances.

**Table 4:** Comparison with previous binocular visual acuities of eyes with AcrySof Vivity intraocular lenses

Study	N	BCDVA, logMAR	BDCIVA, logMAR	BDCNVA, logMAR
Scheepers MA, et al [3]	34	− 0.03 ± 0.05 (6 m)	0.04 ± 0.08 (66 cm)	0.22 ± 0.12 (40 cm)
Kandavel R, et al [10]	32	0.00 ± 0.05 (6 m)	0.12 ± 0.11 (66 cm)	0.36 ± 0.12 (40 cm)
van Amelsfort T, et al [11]	22	− 0.10 ± 0.08 (4 m)	0.03 ± 0.07 (66 cm)	0.28 ± 0.08 (40 m)
Bala C, et al [12]	149	− 0.063 ± 0.092 (4 m)	0.075 ± 0.126 (66 cm)	0.306 ± 0.157 (40 cm)
Current study	24	− 0.15 ± 0.07 (5 m)	0.08 ± 0.12 (66 cm)	0.22 ± 0.16 (40 cm)

Values are presented as means ± standard deviations (distance)

*BCDVA* Binocular corrected distance visual acuity, *BDCIVA* Binocular distance-corrected intermediate visual acuity, *BDCNVA* Binocular distance-corrected near visual acuity, *logMAR* Logarithm of the minimum angle of resolution

A notable outcome of the current study was the distribution of MRSE in the myopic range from –1.20 D to 0.00 D, despite targeting emmetropia. Consequently, the BUCVAs at 66 cm and 40 cm were better than the corresponding BDCVAs, as shown in Fig. 2. As IOL power is available in 0.5 D increments, postoperative refractive outcomes often deviate slightly toward hyperopia or myopia [10]. In a previous study on AcrySof Vivity, postoperative MRSEs ranged from –1.125 to 0.5 D when targeting negative values near zero. Therefore, the slight myopic shift observed in our study is unlikely to be attributable to the material differences between Clareon and AcrySof.

As this study represents the first evaluation of Clareon Vivity IOLs, we assessed their clinical outcomes by aiming for postoperative refraction close to zero in both eyes. Mini-monovision is often used to enhance near vision using EDoF IOLs [15, 16]. In this study, spectacle independence was achieved in 100% of the patients for distance and intermediate vision, and in 62.5% for near vision, similarly to previous mini-monovision results of 90% for distance, 94% for intermediate, and 58% for near vision [16]. Achieving the intended refractive difference between the 2 eyes in mini-monovision can be challenging. Once a toric model becomes available, a prospective study with a larger sample size should be conducted to compare visual function and patient satisfaction between mini-monovision and slight myopia in both eyes.

This study has some limitations. First, the sample size was relatively small. Because toric models are not yet approved, inclusion was restricted to patients with preoperative corneal astigmatism of 0.75 D or less in both eyes. Therefore, future studies with larger sample sizes are necessary once toric Clareon Vivity IOLs become available. Second, the follow-up period was limited to 3 months. As Clareon lenses are expected to maintain a lower incidence of glistening over the long term, further research is required to evaluate lens clarity beyond the short-term period.

In conclusion, bilateral implantation of a nondiffractive EDoF IOL provided good binocular visual function between far and intermediate distances, with minimal photic phenomena.
